# Micro-structural investigations on oppositely charged mixed surfactant gels with potential dermal applications

**DOI:** 10.1038/s41598-021-94777-2

**Published:** 2021-07-30

**Authors:** Manas Barai, Emili Manna, Habiba Sultana, Manas Kumar Mandal, Kartik Chandra Guchhait, Tuhin Manna, Anuttam Patra, Chien-Hsiang Chang, Parikshit Moitra, Chandradipa Ghosh, Anna-Carin Larsson, Santanu Bhattacharya, Amiya Kumar Panda

**Affiliations:** 1 Department of Chemistry, Vidyasagar University, Midnapore, 721102 West Bengal India; 2 Centre for Life Sciences, Vidyasagar University, Midnapore, 721102 West Bengal India; 3 Department of Human Physiology, Vidyasagar University, Midnapore, 721102 West Bengal India; 4 Chemistry of Interfaces Group, Luleå University of Technology, 97187 Luleå, Sweden; 5 Department of Chemical Engineering, National Cheng Kung University, Tainan, Taiwan; 6 India and School of Applied & interdisciplinary Sciences, Indian Association for the Cultivation of Science, Kolkata, 700032 India; 7 Department of Organic Chemistry, Indian Institute of Science, Bangalore, 560012 Karnataka India

**Keywords:** Biophysical chemistry, Drug delivery, Imaging studies, Biomedical materials, Drug delivery, Biomaterials, Liquid crystals, Soft materials, Health care

## Abstract

Dicarboxylic amino acid-based surfactants (*N*-dodecyl derivatives of -aminomalonate, -aspartate, and -glutamate) in combination with hexadecyltrimethylammonium bromide (HTAB) form a variety of aggregates. Composition and concentration-dependent mixtures exhibit liquid crystal, gel, precipitate, and clear isotropic phases. Liquid crystalline patterns, formed by surfactant mixtures, were identified by polarizing optical microscopy. FE-SEM studies reveal the existence of surface morphologies of different mixed aggregates. Phase transition and associated weight loss were found to depend on the composition where thermotropic behaviours were revealed through combined differential scanning calorimetry and thermogravimetric studies. Systems comprising more than 60 mol% HTAB demonstrate shear-thinning behaviour. Gels cause insignificant toxicity to human peripheral lymphocytes and irritation to bare mouse skin; they do not display the symptoms of cutaneous irritation, neutrophilic invasion, and inflammation (erythema, edema, and skin thinning) as evidenced by cumulative irritancy index score. Gels also exhibit substantial antibacterial effects on *Staphylococcus aureus*, a potent causative agent of skin and soft tissue infections, suggesting its possible application as a vehicle for topical dermatological drug delivery.

## Introduction

Formation of gels and different liquid crystalline phases by oppositely charged mixed surfactant systems depend on the composition, surfactant chain length, salinity, temperature, pH and external field, etc.^1–5^. Artificial gels possess regulated super-structure^6–10^, where the properties of the fabricated liquid crystals depend on electrostatic, hydrogen bond, hydrophobic, and van der Waals interactions among the components^11–13^. Gels are associated with two independent transitions, viz*.,* the sol–gel transition of the gelator and anisotropic-isotropic transition of the liquid crystals^9,10,14–18^. Gelatinous property, structure, and shape of surfactant aggregates largely depend on the molecular architecture of the aggregating species^14,19,20^.

Gels have versatile applications in tissue engineering^21^, hemostasis bandages^22–26^, photo-patterning^17,27–30^, 3D-printing^31,32^, electrochemistry^33^, pharmaceutical formulation^5,34–36^, and regenerative medicine^10,37–39^, etc. Recent advances in the design and synthesis of dicarboxylic amino acid-based surfactants (AAS) have opened up their wide range of applications as chelator in metal extraction^40^. Due to its “green nature”, aggregation behaviour of AAS in combination with HTAB have been studied in detail where some mixed surfactants can form gel^16^. This has encouraged the present research group to undertake further investigations on such aggregates at higher concentrations to explore the possibility of using those for topical dermatological drug delivery.

The main aim of the present work is to undertake physicochemical investigations on different types of aggregates formed by AAS + HTAB. While HTAB shows antimicrobial activities, AASs are biocompatible^41^. Because of its toxicity, individual use of HTAB is unwarranted. It is believed that, when HTAB is used in combination with AASs, its toxicity will be substantially reduced^42,43^. To check the biocompatibility of gels and their possible dermatological application in the topical form, cytotoxicity, skin irritation, and histological studies were carried out. HTAB is known to have antimicrobial activities^42^, for which antibacterial activities of AAS + HTAB mixtures are considered to be worth investigating. Hence, antibacterial activities of the gels on *Staphylococcus aureus*, one of the causative agents  for persistent skin and soft tissue infections, were also explored.

## Results and discussion

**Figure 1:**
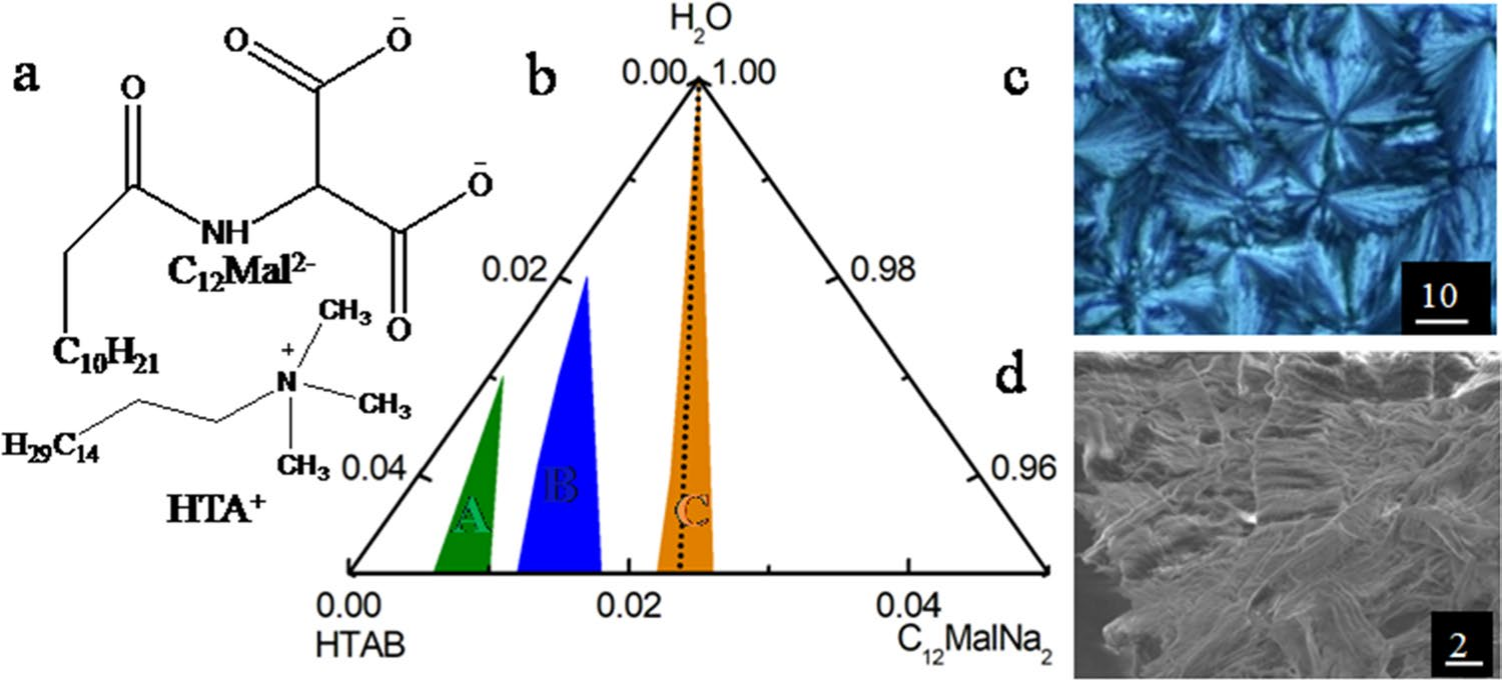
(**a**) Chemical structure of C_12_Mal^2–^ and HTA^+^; (**b**) truncated phase diagram of C_12_MalNa_2_ + HTAB + water mixed system at 25 °C. Phases: (A), gel; (B), viscous; (C), precipitate; and clear region indicate the formation of micelle. The dotted line in panel b corresponds to equimolar region. Panel (**c**), POM and (**d**), FE-SEM image of 5 wt% C_12_MalNa_2_ + HTAB (40:60, *w*/*w*) gel respectively. Scale bars (in μm) are mentioned in the microscopic images.

Structures of C_12_MalNa_2_ and HTAB are shown in (Fig. 1a) along with other information. Manifestation on the Gibbs ternary phase diagram (Fig. 1b) demonstrates the occurrence of gel, viscous, precipitate and clear fluid states. With increasing proportion of HTAB, surfactant mixtures form gels where the relative proportion of viscous and gel states increase following the order: C_12_MalNa_2_ + HTAB > C_12_AspNa_2_ + HTAB > C_12_GluNa_2_ + HTAB (Fig. S1, supplementary section). Hydrophobic interaction between AASs and HTAB is the predominant factor for the formation of different types of aggregates besides the electrostatic attraction^8^. AASs interact with HTAB at a 1:2 mol ratio and form gels at equimolar region due to the dominance of the HTAB molecules^44^. Microstructural investigations on surfactant mixtures at different concentrations (AAS + HTAB) and different compositions (AAS/HTAB) were further investigated through polarizing optical microscopy (POM) and field emission scanning electron microscopic (FE-SEM) studies. POM studies reveal the occurrence of liquid crystal and associated textures as shown in Fig. 1c. Gels exhibit different textures in the surfactant concentration range of 3–5 wt%. With increasing proportions of HTAB, C_12_MalNa_2_ + HTAB gels display nematic, smectic, spherulite, cholesteric, calamitic, and flower-like textures (Fig. S2, Table S1A)^44–47^. Nematic liquid textures originate from thread-like shapes, that correspond to surfactant gels aligning themselves in threadlike shapes as reported earlier^48^. The patterns become more complex with enhanced sizes due to the aggregation and associative interactions between AASs and HTAB. Texture size increases with increasing proportions of HTAB, which has a higher cross-sectional area than the AASs^49^.

Features become more prominent with increasing mixed surfactant concentrations, common in the case of lyotropic liquid crystals^16,47^ (Fig. S2, Supplementary Section). In the case of C_12_AspNa_2_ + HTAB gels for 50, 60, and 80 wt% of HTAB, smectic (Fig. S3a_1_), spherulite (Fig. S3b_1_), and flower-like (Fig. S3c_1_) patterns are observed^50,51^, while in the case of C_12_GluNa_2_ + HTAB mixtures, discotic (Fig. S3a_2_), calamitic (Fig. S3b_2_), and flower-like (Fig. S3c_2_, Table S1B) textures are observed due to the formation of sterically favourable seven and eight-member rings. Two carboxylate groups get progressively separated by one methylene group while moving from C_12_MalNa_2_ to C_12_AspNa_2_ to C_12_GluNa_2_. Accordingly, two carboxylate groups of AASs can electrostatically interact with one HTAB to form six, seven, and eight-member rings^49^. Smectic textures designate ordered and rigid layer structure whereby C_12_MalNa_2_ + HTAB can closely interact with HTAB to exhibit smectic textures (Fig. S2b_1_,c_2_,a_4_,c_4_). The nematic texture (Fig. S2a_1_,b_2_,a_3_), is characteristic of stacked layer and positional order whereby the discotic texture (Fig. S2b_4_) is due to rigid disk-like core, according to Fan Shao et al.^52,53^. Spherulite textures (Fig. S2a_5_ and b_3_) are larger bundles and the hexagonal shapes caused by close packing are even more defined, as also reported by Haas et al.^54^ C_12_MalNa_2_ + HTAB gels display a more prominent spherulite texture (Fig. S2a_5_,b_3_) than C_12_AspNa_2_ + HTAB due to the formation of strongly aggregated structure and associative interaction with HTAB. Calamitic textures, exhibited by C_12_GluNa_2_ + HTAB gels (Fig. S3b_2_, Table S1B) have a relatively flexible core, due to weak hydrophobic interaction between the oppositely charged C_12_GluNa_2_ and HTAB system.

Microstructures of the aggregates were further investigated with FE-SEM studies, which display interconnected morphologies (Fig. 1d)^27,55,56^. To achieve optimal solvation and swelling, the pore of the gels can provide pockets for water molecules, necessary for hydration, to be included by surface tension. C_12_MalNa_2_, in combination with HTAB, shows flower-like (Fig. S4e_1_,b_4_,b_5_,c_5_,e_5_ and Table S1A), coral-like (Fig. S4a_1_,d_2_), and porous—(Fig. S4a_3_) architectures due to the existence of protrusions and larger channels^57^. Flake—(Fig. S4c_1_,b_3_,c_4_), leaf—(Fig. S4b_1_,d_1_,a_2_), leaf + flake—(Fig. S4b_2_), wrinkled—(Fig. S4a_5_), and sheet-like structures (Fig. S4d_3_) display extended flat features. Fibrous texture (Fig. S4c_3_ and d_5_) indicate larger bundled-fibre network structure. Granular—(Fig. S4e_2_,e_4_) and cuboid (Fig. S4e_3_) morphologies were observed in some cases. Irregular structure (Fig. S4c_2_) and amorphous materials (Fig. S4a_4_,d_4_) had also been observed, in which cases the aggregates do not have any particular features. In the cases of C_12_AspNa_2_ + HTAB gels with 50, 60, and 80 wt% of HTAB exhibits fibrous, dense fibrous (Fig. S5a_1_,b_1_, and Table S1B) and densely-packed cuboid structures (Fig. S5c_1_). C_12_GluNa_2_ + HTAB mixtures exhibit cuboid (Fig. S5a_2_), irregular structures (Fig. S5b_2_), and sheet-like structures (Fig. S5c_2_)^27,28,57–59^. C_12_AspNa_2_ + HTAB and C_12_GluNa_2_ + HTAB gels show characteristic cuboid structures (Fig. S5c_1_,a_2_) due to the emergence of micropores at the surface of gels^60^. C_12_AspNa_2_ + HTAB gels have fibre network-like morphologies (Fig. S5a_1_,b_1_) that can hold water molecules due to assisted surface tension enhancement. Irregular (Fig. S5b_2_) and sheet-like structures (Fig. S5c_2_) for C_12_GluNa_2_ + HTAB indicate the entrapment of water molecules into the gels. Maximum number of HTAB accumulated in gels indicate that HTAB plays a fundamental role in demonstrating higher aggregation and formation of porous-like morphology, which are in consonance with the phase manifestation, and POM studies.

**Figure 2:**
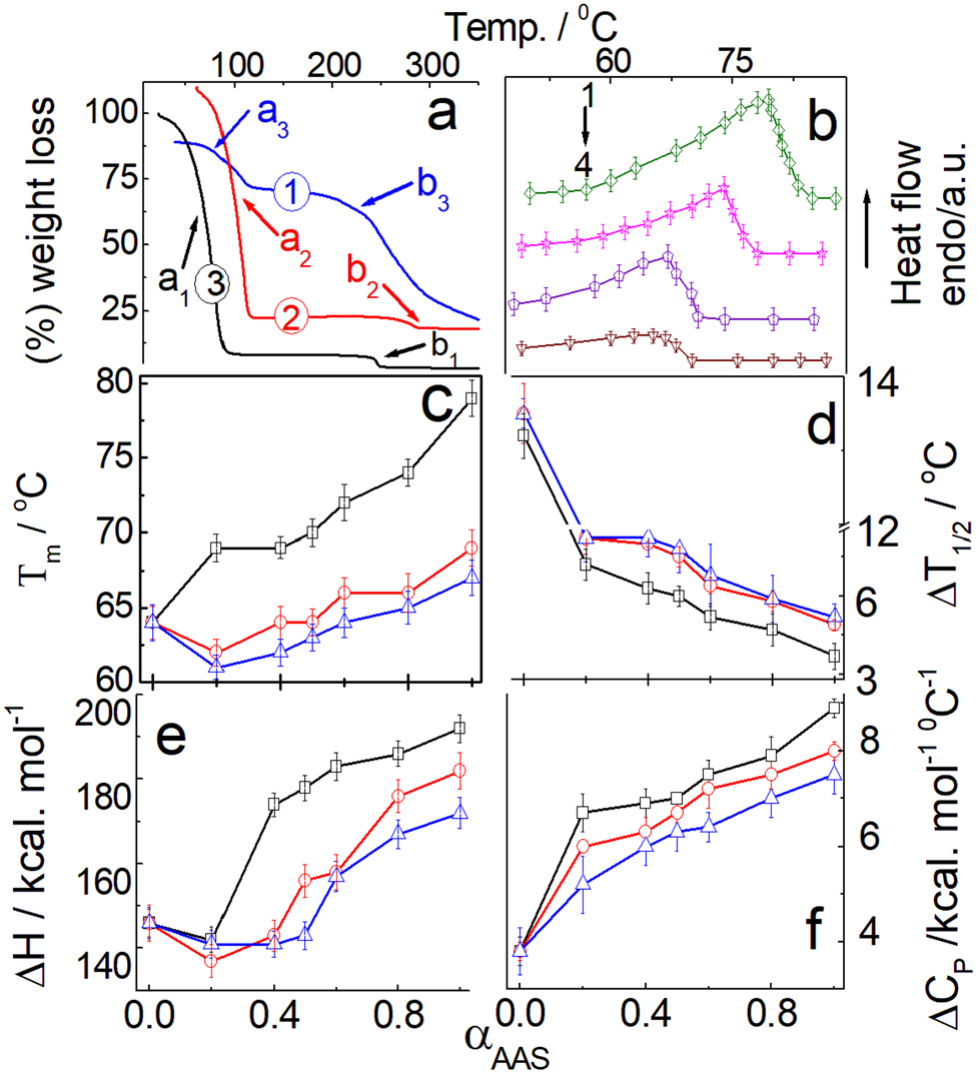
(**a**) TGA of AAS + HTAB (100 mM, 40/60, M/M) gel. Systems: 1, C_12_GluNa_2_ + HTAB; 2, C_12_AspNa_2_ + HTAB and 3, C_12_MalNa_2_ + HTAB. a_1_, b_1_, a_2_, b_2_, a_3_, and b_3_ represent different phase transitions. (**b**) DSC of C_12_MalNa_2_ + HTAB mixture at different mole% of C_12_MalNa_2_: 1, 80; 2, 60; 3, 40 and 4, 0. Variations of (**c**) chain melting temperature (*T*_m_); (**d**) half peak height (Δ*T*_1/2_); (**e**) enthalpy change (Δ*H*) and (**f**) heat capacity change (Δ*C*_p_) with the mole fraction of AAS (*α*_AAS_). The line colours in panels (**c**–**f**) represent similar surfactant composition as in panel (**a**). Scan rate: 2 °C min^−1^.

Phase transition and associated weight loss of gels were investigated by thermogravimetry analysis (TGA)^61–63^. Results on the TGA of the pure components, as well as AAS + HTAB aggregates, have been summarized in Fig. S6 and Table S2. HTAB decomposes to produce some solid carbon along with the production of long-chain hydrocarbon, nitrogen, and hydrogen^61,64^, whereby decomposition of C_12_MalNa_2_, C_12_AspNa_2_ and C_12_GluNa_2_ to produce dodecane (or smaller alkyl fragments) and free aminomalonic, aspartic, and glutamic acid^63^. AAS + HTAB gels show endothermic peaks in the temperature range of 40 to 100 °C due to dehydration (Fig. 2a)^65^. Two carboxylate groups of C_12_MalNa_2_ electrostatically interact with HTAB that result in higher ionicity and subsequent moisture absorption capability than C_12_AspNa_2_ and C_12_GluNa_2_. In the case of C_12_GluNa_2_, two ionic carboxylate groups are separated by three methylene groups; so its interaction capability with HTAB and magnitude of hydration is lower. Formation of rigid aggregates result in the higher chain melting temperature (*T*_m_) as determined from the DSC studies.

Thermotropic behaviour and associated parameters were evaluated by DSC studies^44^. Variation of phase transition temperature (*T*_m_), width at half peak height (Δ*T*_1/2_), enthalpy change (Δ*H*) and corresponding heat capacity changes (ΔCp) were determined as functions of *α*_AAS_ as summarized in (Fig. 2). HTAB exhibits two endothermic peaks at 64 °C and 84 °C indicating transition from solid to liquid crystalline phase and the phenomenon of dehydration^44^, common in the amphiphiles (Fig. 2b)^63^. With increasing *α*_AAS_, *T*_m_ values increase due to the incorporation of HTAB in the aggregates and assimilation of HTAB with AASs; relatively sharp peaks indicate favourable hydrocarbon chain packing (Fig. 2c)^49^. Lowering of *T*_m_ is due to size reduction, decreased specific surface area, and interaction between oppositely charged surfactants also known as Kelvin effect^66^. Widening of the melting peaks designates multi-crystallinity and heterogeneity^66^. The extent of hydrophobic interaction between AAS and HTAB is lower in C_12_AspNa_2_ and C_12_GluNa_2_, where the *T*_m_ values follow the sequence: C_12_MalNa_2_ + HTAB > C_12_AspNa_2_ + HTAB > C_12_GluNa_2_ + HTAB. With increasing *α*_AAS_, Δ*T*_1/2_ values decrease indicating better packing of the hydrophilic overlayer as well as oppositely charged head groups (Fig. 2d). With increasing associative interaction between AAS and HTAB increased crystal imperfection results in higher Δ*T*_*1*/2_ values. Increasing magnitude of interaction with increasing proportion of HTAB induces the formation of ion-pair amphiphiles, resulting in higher Δ*H* values for the surfactant mixtures (Fig. 2e). With increasing *α*_AAS_, Δ*C*p values gradually increase and exhibit endothermicity due to the formation of water overlayer around surfactant aggregates (Fig. 2f). Lower values of Δ*C*p are due to the increase in multicrystallinity.

**Figure 3:**
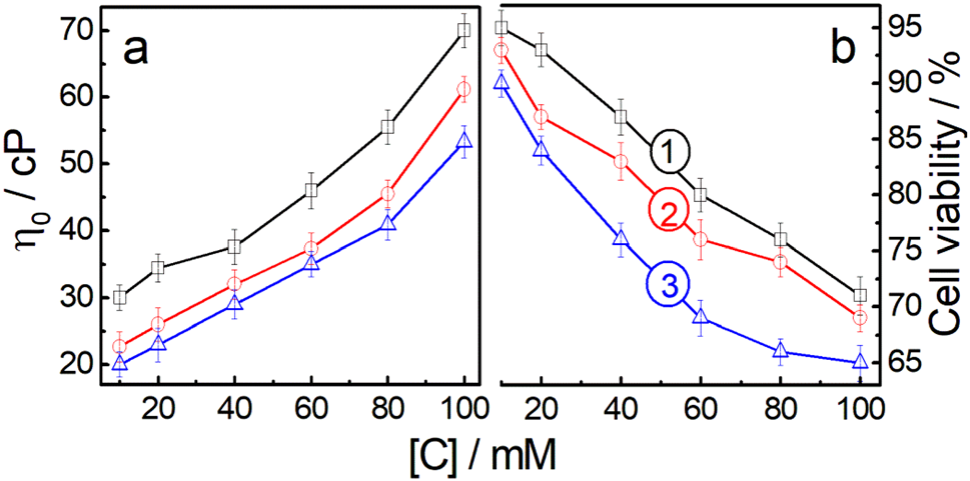
Variation of a, zero shear viscosity and b, human blood lymphocyte cell viability with AASs + HTAB (2/3, M/M) mixed surfactant concentration [C]. Systems: 1, C_12_MalNa_2_ + HTAB; 2, C_12_AspNa_2_ + HTAB and 3, C_12_GluNa_2_ + HTAB.

Viscosity studies on AAS + HTAB mixtures at different combinations exhibit shear-thinning (results not shown)^49^. Zero shear viscosity (*η*_0_) *vs.* concentration profile for the gels comprising 60 mol% HTAB are shown in Fig. 3a.

With increasing surfactant concentrations, viscosity increases monotonously in the range of 10 to 100 mM. The sequence in the viscosity variation follows the order: C_12_MalNa_2_ + HTAB > C_12_AspNa_2_ + HTAB > C_12_GluNa_2_ + HTAB, which are in consonance with the previous studies. Due to the stronger packing and subsequent formation of rigid-structured aggregates, the viscosity of C_12_MalNa_2_ + HTAB system is higher than the other two.

AAS + HTAB aggregates exhibit less toxicity against human blood lymphocyte up to 20 mM; although, cytotoxicity increases with increasing surfactant concentration (Fig. 3b). Results suggest that cytotoxicities are in consonance with the corresponding viscosity of the system.

**Figure 4:**
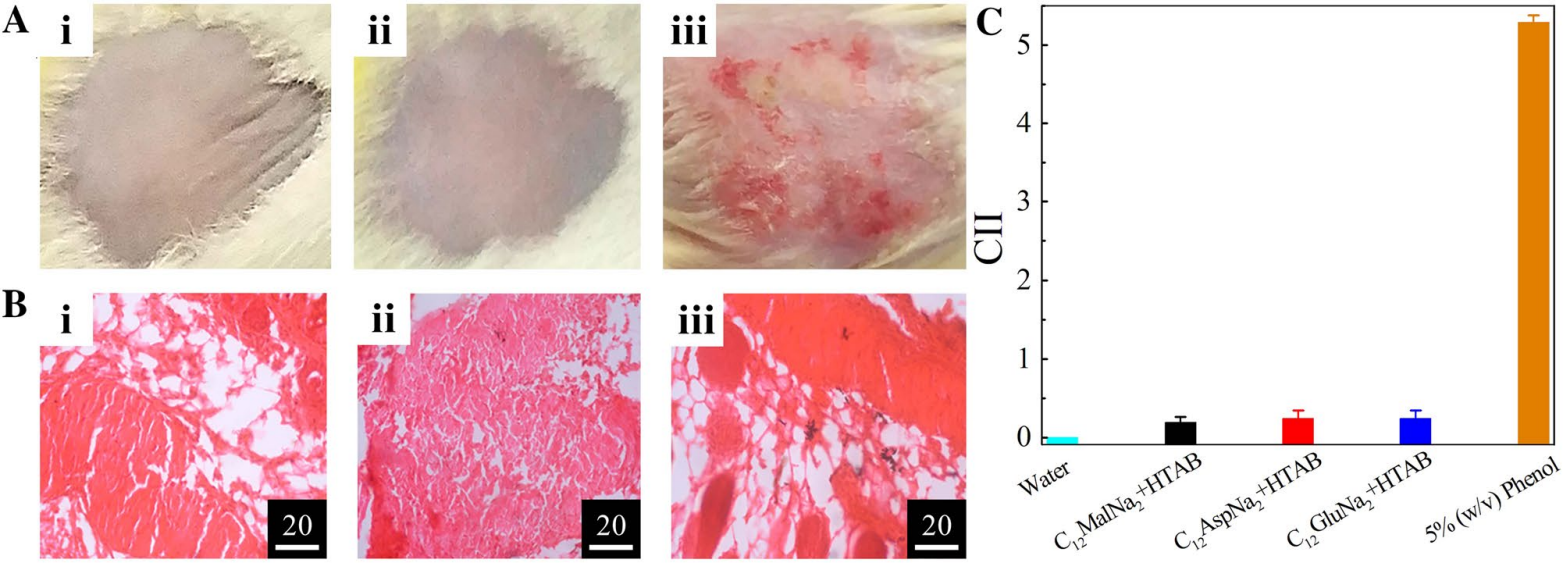
Effects of AAS + HTAB gels on mice skin. Gels were applied at the dorsal area of the trunk region of swiss albino mice for seven consecutive days and the effects recorded are presented through (**A**) photographs of treated skins and (**B**) corresponding histopathological evaluation of inflammation through micrographs. Systems: i, sterile distilled water (NC); ii, C_12_MalNa_2_ + HTAB gel (100 mM, 60/40, M/M) and iii, 5% (*w*/*v*) phenol-water (PC). Micrographic scale bar: 20 μm. (**C**) cumulative irritancy index (CII) scores for skins treated with different systems, calculated according to Organization for Economic Co-operation and Development (OECD) guidelines^67^. Overall comparison of CII scores among the groups were carried out by the Kruskal–Wallis test followed by post-hoc Dunn’s test for multiple comparisons between each pair of groups. Significant differences in CII scores at *P* < 0.05 were obtained between groups PC *vs.* NC, PC *vs.* C_12_MalNa_2_ + HTAB, PC *vs.* C_12_AspNa_2_ + HTAB, and PC *vs.* C_12_GluNa_2_ + HTAB gels.

Dermal responses towards the gels were assessed through topical application of the experimental gels on the bare mice skin surface where sterile distilled water and 5% (*w*/*v*) phenol-water were used as a negative (NC) and positive control (PC), respectively. Dermal irritability, as evidenced by the development of edema, and erythema were tested carefully and were scored according to the Organization for Economic Co-operation and Development (OECD) guidelines﻿ (Fig. 4A,C).

Simultaneously, developments of skin inflammation upon the application of gels were studied histologically by examining the hematoxylin-eosinY stained gel-treated skins for neutrophil invasion (Fig. 4B)^68,69^. It was found that gels do not induce symptoms related to skin irritation and inflammation (erythema, edema, and neutrophilic invasion) as shown in Fig. 4A(ii),B(ii). Effects of gels on mice skin appeared similar to the effect of sterile distilled water, used as negative control (NC). On the other hand, 5% (*w*/*v*) phenol-water (PC) caused considerable erythema, edema, and neutrophilic invasion Fig. 4A(iii),B(iii), resulting in high CII score (> 5.00) that corresponds to moderate skin irritation^67^. The CII score, for each of the gel treatment, was calculated to be as low as < 0.49 (Fig. 4C). These values, along with the CII values observed in NC group, correspond to the scores representing no potential skin irritation according to the OECD guidelines^67^. Besides, mice treated with 5% (*w*/*v*) phenol-water (PC group) visibly suffered from post-treatment erythema and edema resulting in higher CII score (> 5.00) that corresponds to moderate skin irritation^67^. Similarly, Fig. 4C reveals that there were significant differences (*P* < 0.05) in CII scores between 5% (*w*/*v*) phenol-water-treated group and each of the gel-treated groups. Based on these findings, it could be concluded that gels used in this study do not have significant skin irritation potential and thus may safely be used for topical dermal applications.

Gels also exhibited substantial antibacterial activities against *Staphylococcus aureus*, a gram-positive pathogenic bacteria. In spite of ~ 90% lymphocyte viability, 20 mM surfactant mixtures of AAS + HTAB (40:60, M/M) could substantially inhibit bacterial growth^67^. The minimum inhibitory concentration (MIC) is the lowest concentration of a substance that renders no turbidity in bacterial culture, corresponding to 99% bacterial growth inhibition^70^. The MIC value of individual surfactant mixture was determined for different clinical isolates of *S. aureus,* *i.e.*, American Type Culture Collection (ATCC) 25923 and four other clinical isolates: AK1, AK2, AK8, and AK10 with the broth dilution method. Vancomycin was also used as a reference drug for growth inhibition^71,72^ as it is a widely used effective ‘drug of choice’ against *S. aureus* infections^73^. With the MIC value in the range of 10–40 µM, C_12_MalNa_2_ + HTAB and C_12_GluNa_2_ + HTAB gels exhibited higher antibacterial activities than C_12_AspNa_2_ + HTAB, with the MIC value in the range of 20–60 µM, as summarized in Table 1.

**Table 1:** Antibacterial activities of AAS + HTAB gels against *S. aureus* clinical isolates.

*S.aureus* strains	MIC (µM)^a^			
	Vancomycin	C_12_MalNa_2_ + HTAB	C_12_AspNa_2_ + HTAB	C_12_GluNa_2_ + HTAB
AK1	0.827	20	40	20
AK2	1.242	40	60	40
AK8	1.035	40	50	40
AK0	0.827	30	40	30
ATCC 25923	0.482	10	20	10

^a^For the determination of MIC value, serial concentrations in the range of 5 to 100 µM (with an increment of 5 μM in each step) of each surfactant mixture was applied to bacterial culture containing 1 × 10^7^ CFU. Data represent mean values of three experimental outcomes.

Results indicate that gels can potentially be used in the treatment of bacteria-borne dermatological infections. In addition, the gels are expected to exhibit higher entrapment efficiency and sustained release of dermatological drugs in terms of topical applications. However, further in vitro and in vivo studies are warranted to substantiate the aptitude of the gels as potential drug delivery systems. Besides, studies involving composition structure and functional correlationships are also considered to be worthy.

## Summary and conclusions

Microstructures of AAS + HTAB aggregates were investigated by combined phase manifestation, polarizing optical microscopy and field-emission scanning electron microscopic studies. Texture of liquid crystals depended on the concentration and proportion of constituent surfactants. Energetics of phase transition processes were evaluated by TGA and DSC studies. Cytotoxicity could be correlated with the viscosity of the gels. Gels impart insignificant skin irritation although they possess substantial antibacterial activities that project their potential as dermal drug delivery systems. However further in vitro and in vivo studies by incorporating appropriate drugs into the gels are necessary and is considered as the future perspectives.

In order to draw final conclusions about solvation, water channels, and pores, drawn from the morphological studies, further characterization techniques, viz*.,* small-angle X-ray scattering and small-angle neutron scattering studies, combined with the molecular dynamics simulation studies are worthy to be investigated. These are considered as the future perspectives.

## Experimental section

### Materials

Hexadecyltrimethylammonium bromide (HTAB), fetal bovine serum (FBS) and histopaque-1077™ were the products from Sigma-Aldrich Chemicals Pvt. Ltd. (USA). Disodium salts of AAS were synthesized according to the previous reports^74^. Phenol, hematoxylin, eosin Y, and 3-(4, 5-dimethylthiazol-2-yl)-2, 5-diphenyl tetrazolium bromide (MTT) were purchased from Hi Media Laboratories Pvt. Ltd. India. Double distilled water was used throughout the experiments. All the chemicals were stated to be ≥ 99% pure and were used as received.

### Methods

#### Phase manifestation

Composition, close to the boundary of the two-phase regions, was detected by homogeneous mixing of aqueous stock solution of oppositely charged surfactants at 25 °C. The exact boundary of the two-phase region was detected by further step wise addition (using a calibrated micropipette under constant stirring) of a higher concentration of AASs into the HTAB solution. On the basis of visual observation, more than one hundred samples were collected at different AASs and HTAB weight ratios and phase boundaries were identified^75^. The different phases were recorded consecutively for a longer time-period (upto fifteen days, after which the samples started microbial degradation). All the experiments were repeated thrice to ensure reproducibility.

#### Microscopic studies

The texture of different combinations of the mixed surfactant systems were recorded with a polarizing optical microscope (POM, Nikon ECLIPSELV100POL, Japan) set with a CCD camera. The sample was placed onto a glass slide and thereafter the POM images were recorded. Morphology of the surfactant aggregates were investigated with field emission-scanning electron microscopy (FE-SEM, ZEISS EVO 18, Germany). Samples were prepared by the drop-casting of the gel on a freshly cleaved mica foil and kept in air for two hours for solvent evaporation. Those were further dried at reduced pressure for two hours. The gold-sputtered samples were then analysed in FE-SEM at the operating voltage of 20–30 kV.

#### Thermogravimetric analysis (TGA)

Weight loss and thermal stability of the gels were investigated by TGA, performed using Pyris 6 TGA-DTA-8000 (Perkin Elmer, USA). Samples were scanned in the temperature range of 50–500 °C with a scan rate of 2 °C min^−1^ under nitrogen gas flowing conditions.

#### Differential scanning calorimetric (DSC) studies

DSC studies were performed to evaluate the chain melting temperature (*T*_m_) and associated thermodynamic parameters of mixed surfactant systems that control its physical states. DSC measurements were recorded using a Pyris 6 DSC-8000 (Perkin Elmer, USA) differential scanning calorimeter with indium as a calibrator before performing the experiment. After equilibrating for 10 min, the sample was scanned in the temperature range 0–100 °C with a scan rate of 2 °C min^−1^ during the heating cycle. From the thermogram the peak temperature and enthalpy of phase transition were evaluated. Endothermic peak *vs.* temperature in evaluating different physicochemical parameters of mixed surfactant system were considered^76^.

#### Rheology studies

Viscosities of different surfactant mixtures were determined with a DV II-Pro rotoviscometer (Brookfield, USA) with a stated accuracy of ± 0.01 cP. 1.0 mL surfactant solution of different concentrations (40, 60, 80, and 100 mm) were taken in a cone and plate type rotoviscometer separately^77^. Viscosities were measured at different shear rates (ranging from 76 to 380 s^−1^). Zero shear viscosity (*η*_0_) was determined from the intercept of the plot of viscosity *vs.* shear rate by fitting polynomial regression. Temperature during the viscosity measurement was controlled by a circulatory water bath MIC-255 (Hanntech Corporation, South Korea).

#### Biological activities

All the biological experiments were performed in accordance with relevant guidelines and regulations, duly approved by the Institutional Ethics Committee, Vidyasagar University. All the methods were carried out in accordance with relevant guidelines and regulations.

#### Cytotoxicity studies

Cytotoxicity studies were carried out following the method of Sun et al.^67^ 5 mL of human blood (volunteered by healthy persons) was diluted (1:1) with phosphate-buffered saline (PBS) and added to Histopaque-1077. Informed consent was obtained from all subjects. It was centrifuged at 1500 rpm for 40 min at the room temperature. The upper layer containing lymphocytes was further washed through centrifugation. Lymphocytes were re-suspended in Roswell Park Memorial Institute (RPMI) complete media supplemented with 10% (*w/v*) FBS and incubated for a day at 37 °C in 5% (*v/v*) CO_2_ environment (in CO_2_ incubator)^78^. Cytotoxicity of selected gels were estimated with MTT assay^79^. 20 μL 5% (*w*/*v*) MTT solution was added to each well of the microtitre plate, having RPMI-suspended lymphocytes with or without the gels. Then the plate was incubated at 37 °C for 4 h in metabolizing MTT to formazan. After the aspiration of the supernatant, 100 μL HCl + isopropanoic acid solution (1:1) was added to each well of the culture plate and mixed to dissolve the formazan crystals. Optical density (OD) of the sample was measured on an ELISA reader (Model 550, BIO-RAD, USA) using test and reference wavelengths of 570 and 630 nm, respectively. Percentage of cell viability was calculated using the following equation^79^:

1 $${\text{Cell }}\;{\text{viability }}\;\% \, = \, \left[ {{\text{OD}}_{{{\text{sample}}}} {-}{\text{ OD}}_{{{\text{control}}}} } \right] \, \times { 1}00/{\text{OD}}_{{{\text{control}} }}$$

#### Skin irritation test

The biocompatibility of the experimental gels (AAS + HTAB) were investigated through skin irritation tests on swiss albino mice, in compliance with the Animal Research: Reporting of in vivo Experiments (ARRIVE) guidelines^80^. The tests were performed following the method mentioned in the Good Laboratory Practice Standards (GLPS) manual and the guidelines of OECD for acute dermal irritation^81^. Thirty healthy swiss albino mice were divided into five groups, each group consisting of six mice. Group A was negative control (NC, treated with sterile distilled water) and group B was positive control (PC, treated with 5% (*w*/*v*) phenol-water). Mice of group C, D, and E were treated individually with 100 mM C_12_MalNa_2_ + HTAB, C_12_AspNa_2_ + HTAB and C_12_GluNa_2_ + HTAB gels, respectively. Prior to the application of gels, hairs of the dorsal area of the trunk region of all mice were removed followed by the topical application of 500 mL of the gels and the controls, respectively. After 1 h, signs of erythema or edema in individual animal was recorded. The entire procedure of dermal application of gels and subsequent recording for any irritation was continued for seven consecutive days. The CII score for each of the treated groups were calculated according to OECD guidelines^81^. CII score of each treated group is the average score, i.e., score of a total of erythema and edema divided by the number of animals and testing days.

#### Histological studies of mice skin

Animals were sacrificed post euthanasia by carbon dioxide asphyxiation after the completion of skin treatment with gels and controls for seven consecutive days. The treated skins were processed in wax blocks and transverse sections were prepared, followed by staining with hematoxylin-eosinY (HE)^69^. The prepared skin sections were further examined under a light microscope (Axioscope A1; Carl Zeiss, Germany). Histology of the gel-treated skins were compared with the controls.

#### Studies on antibacterial activity

Antibacterial efficacies of the gels against the gram-positive bacterial pathogen, *Staphylococcus aureus*, grown in Luria Bertani (LB) broth, were evaluated. MIC of each gel against the five clinical isolates of *S. aureus* (AK1, AK2, AK8 and AK10 along with ATCC 25923) was determined by broth dilution method. 10 µL gelatinous suspension of surfactant mixture at particular composition was added to 1 mL bacterial culture in LB having approximately 1 × 10^7^ CFU. Each surfactant mixture (AAS + HTAB) was added to the bacterial culture in the concentration range of 5 to 100 μM (with an increment of 5 μM in each step) in a serial manner for individual bacteria and were incubated at 37 °C for 18 h^70–72,82^. Vancomycin was used as reference drug for growth inhibition. All the experiments were repeated thrice.

#### Statistical analysis

The mean value and standard deviation of the quantitative variables were calculated after repeating each quantification three times. Overall comparisons of data among the groups were carried out by the Kruskal–Wallis test. This was followed by post hoc Dunn’s test for multiple comparisons of data between each pair of groups. Differences were considered significant at *P* < 0.05^83^.

## Supplementary Information

Supplementary Information.
