# Supplementary material for: Micro-structural investigations on oppositely charged mixed surfactant gels with potential dermal applications
Source: Sci Rep. 2021 Jul 30;11:15527. doi: 10.1038/s41598-021-94777-2 (PMC8324821; doi:10.1038/s41598-021-94777-2)
Supplement: Supplementary file 1 — Supplementary Information. [file 41598_2021_94777_MOESM1_ESM.docx]

**Micro-structural investigations on oppositely charged mixed surfactant gels with potential dermal applications**

Manas Barai,^1^ Emili Manna,^2^ Habiba Sultana,^1^ Manas Mandal,^1^ Kartik Chandra Guchhait,^3^ Tuhin Manna,^3^ Anuttam Patra,^4^ Chien-Hisang Chang,^5^ Parikshit Moitra,^6^ Chandradipa Ghosh,^3^ Anna-Carin Larsson,^4^ Santanu Bhattacharya^6^ and Amiya Kumar Panda^1,*^

^1^Department of Chemistry, Vidyasagar University, Midnapore - 721102, West Bengal, India

^2^Centre for Life Sciences, Vidyasagar University, Midnapore-721102, West Bengal, India

^3^Department of Human Physiology, Vidyasagar University, Midnapore - 721102, West Bengal, India

^4^Chemistry of Interfaces Group, Luleå University of Technology, SE-97187, Luleå, Sweden

^5^Department of Chemical Engineering, National Cheng Kung University, Tainan, Taiwan

^6^Indian Association for the Cultivation of Science, Jadavpur, Kolkata-700032, India










**Fig. S1.** Variation in relative area of precipitate, viscous, and gel phase of (a), C_12_MalNa_2_+HTAB; (b), C_12_AspNa_2_+HTAB and (c), C_12_GluNa_2_+HTAB mixture at different surfactant concentrations. Temperature: 25^°^C.

**
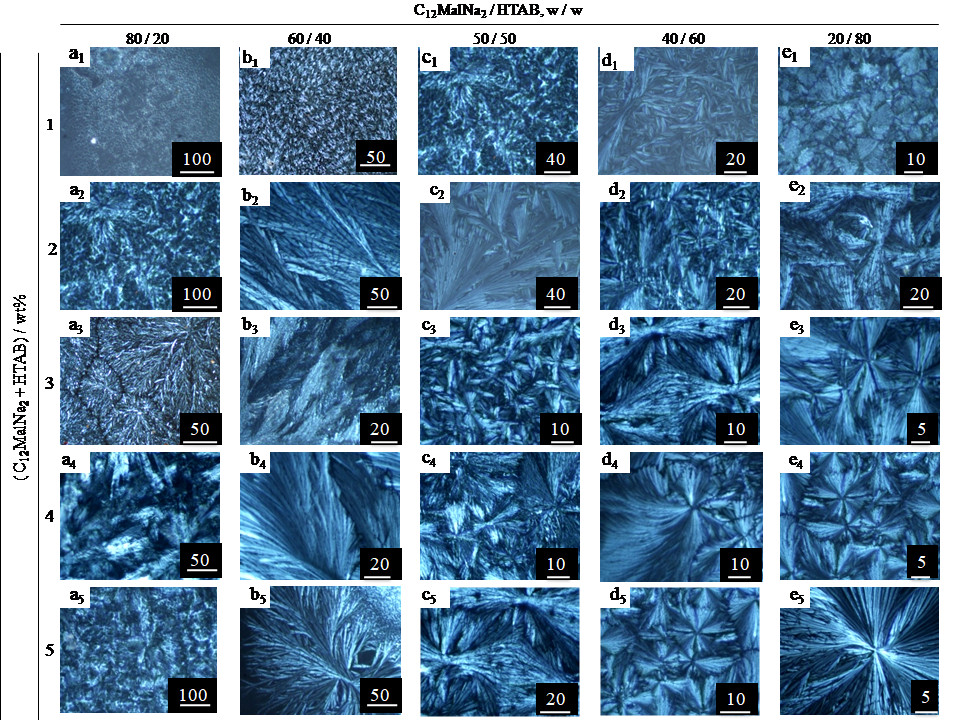
**

**Fig. S2.** POM images of C_12_MalNa_2_ + HTAB mixtures at different compositions. (C_12_MalNa_2_+ HTAB) concentration (wt %): a_1_, a_2_, a_3_, a_4_, and a_5_: 1, 2, 3, 4, 5; C_12_MalNa_2_: HTAB (*w*/*w*): a_1_, b_1_, c_1_, d_1_, and e_1_: 80/20, 60/40, 50/50, 40/60, and 20/80 *w*/*w*. Scale bars in (µm) are shown inside the images.


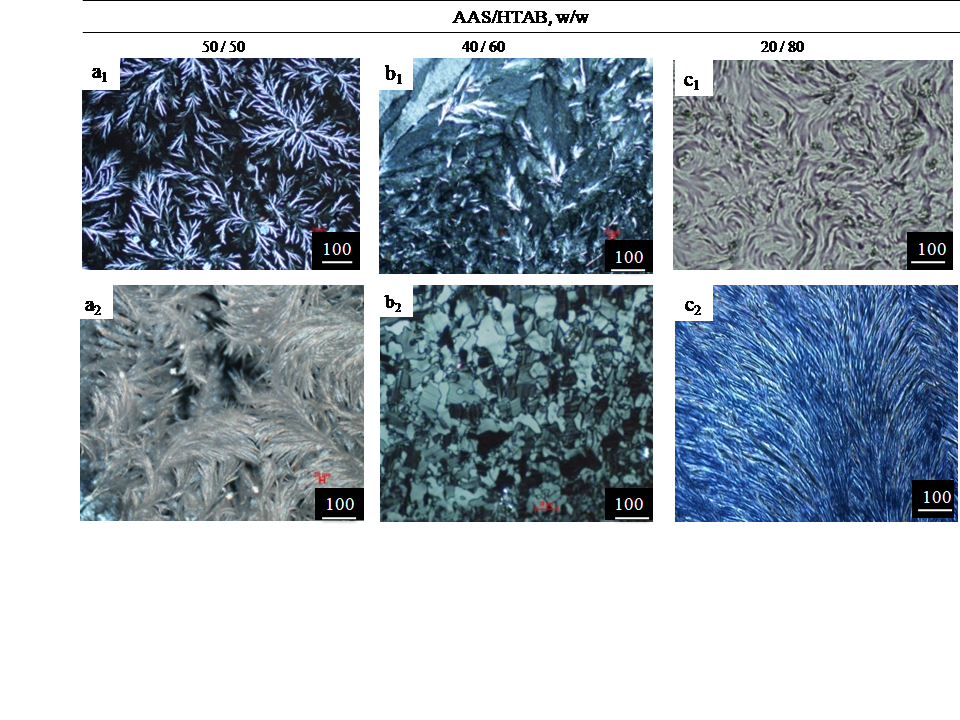


**Fig. S3.** POM images of (C_12_AspNa_2_+ HTAB; a_1_, b_1_, and c_1_) and (C_12_GluNa_2_+ HTAB; a_2_, b_2_, and c_2_) at 5 wt% of total surfactant concentration and at different (50/50, 60/40, and 80/20) weight ratio of AAS/HTAB. Scale bars in (µm) are shown inside the figure.


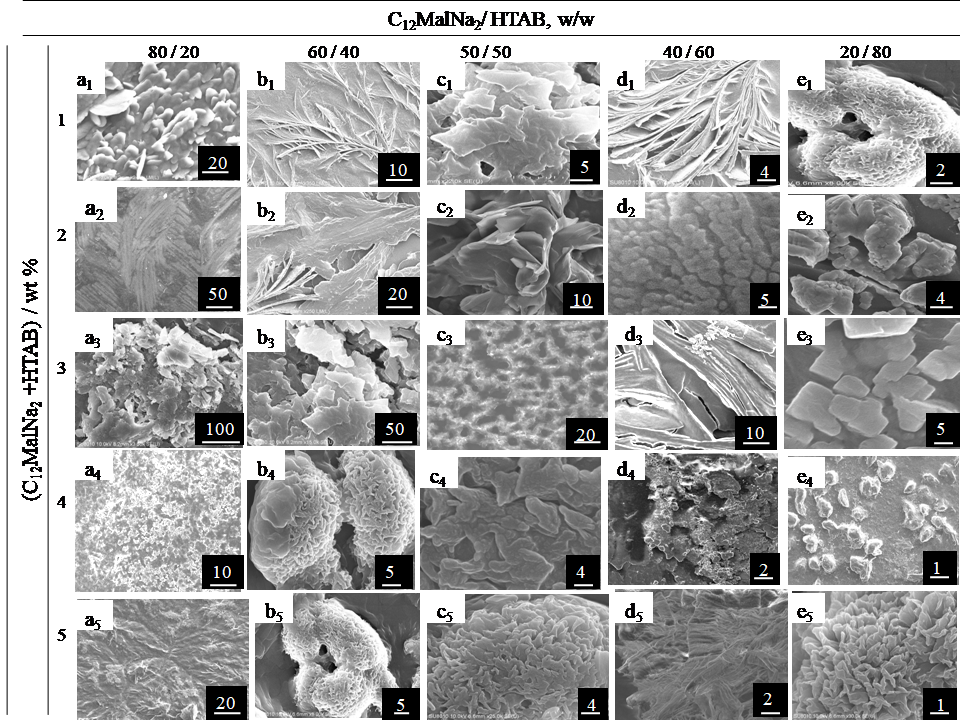


**Fig. S4.** FE-SEM images of C_12_MalNa_2_+HTAB at different concentration (wt%): a_1_, a_2_, a_3_, a_4_, and a_5_: 1, 2, 3, 4 and 5 at the different C_12_MalNa_2_/HTAB ratio (*w*/*w*): a_1_, b_1_, c_1_, d_1_, and e_1_: 80/20, 60/40, 50/50, 40/60, and 20/80*w*/*w*. Scale bars in (µm) are shown inside the images.

**
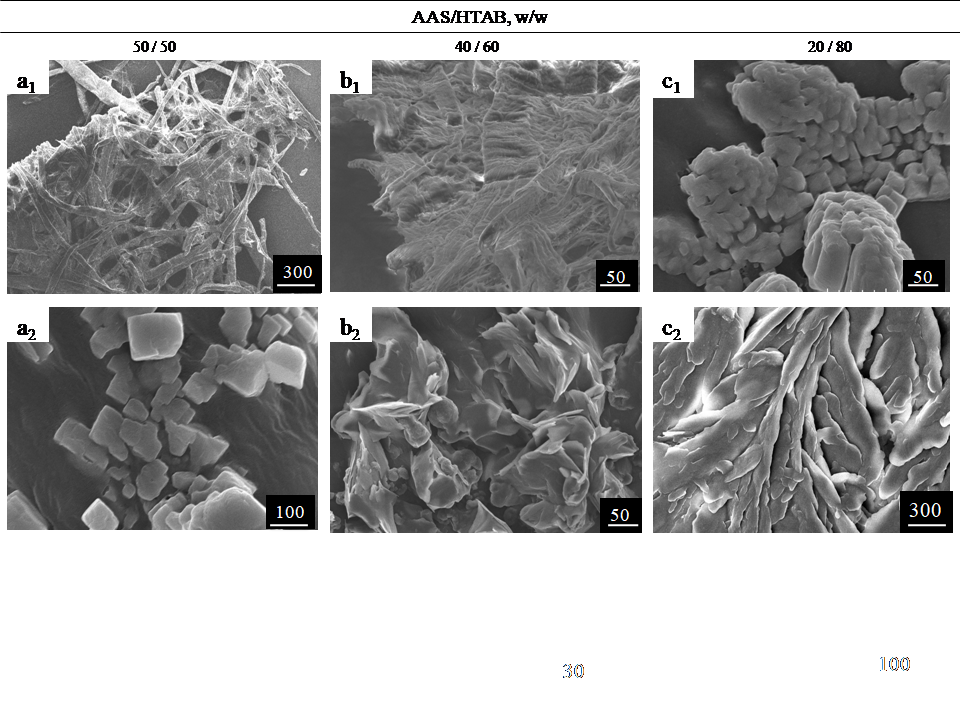
**

**Fig. S5.** FE-SEM images of C_12_AspNa_2_+HTAB (a_1_, b_1_, and c_1_) and C_12_GluNa_2_+HTAB (a_2_, b_2_, and c_2_) at 5 wt% of total surfactant concentration and at different (50/50, 60/40, and 80/20) weight ratio of AAS/HTAB. Scale bars in (µm) are shown inside the figure.

**Table S1A.** Summarized results on the POM (Corresponding to Figure S2) and FE-SEM (Corresponding to Figure S4) studies at different wt% of C_12_MalNa_2_+HTAB aggregates.

| **(C_12_MalNa_2_+HTAB wt%)** | **C_12_MalNa_2_/ HTAB (*w*/*w*)** | | | | |
| --- | --- | --- | --- | --- | --- |
|  | 80/20 | 60/40 | 50/50 | 40/60 | 20/80 |
|  | (a) | (b) | (c) | (d) | (e) |
| **Type of the texture/ liquid crystal** | | | | | |
| 1 | nematic | smectic | columnar | rod | disk |
| 2 | calamitic | nematic | smectic | columnar | flower |
| 3 | nematic | spherulite | cholesteric | flower | flower |
| 4 | smectic | discotic | smectic | flower | flower |
| 5 | spherulite | flower | calamitic | flower | flower |
|  | **SEM (morphology)** | | | | |
| 1 | coral | leaf | flake | leaf | flower |
| 2 | leaf | leaf+flake | irregular structure | coral | granular |
| 3 | porous | flake | fibrous network | sheet-like structure | cuboid |
| 4 | amorphous | flower | flake | amorphous | granular |
| 5 | wrinkled | flower | flower | dense fibrous | flower |

**Table S1B.** Summarized results on the POM (Corresponding to Figure S3) and FE-SEM (Corresponding to Figure S5) images at 5 wt% of total surfactant concentration and at different wt. ratio of C_12_AspNa_2_+HTAB and C_12_GluNa_2_+HTAB aggregates.

| System |  | **AAS/HTAB (*w*/*w*)** | | |
| --- | --- | --- | --- | --- |
|  |  | 50/50 | 40/60 | 20/80 |
|  |  | (a) | (b) | (c) |
|  |  | **Type of the texture/ liquid crystal** | | |
| (C_12_AspNa_2_ + HTAB) | 1 | smectic | spherulite | flower |
| (C_12_GluNa_2_ + HTAB) | 2 | discotic | calamitic | flower |
|  |  | **SEM (morphology)** | | |
| (C_12_AspNa_2_ + HTAB) | 1 | fibrous | dense fibrous | densely-packed cuboid |
| (C_12_GluNa_2_ + HTAB) | 2 | cuboid | irregular structure | sheet-like structure |





**Fig. S6.** TGA of AASs and HTAB. Systems: 1, C_12_MalNa_2_; 2, C_12_AspNa_2_; 3, C_12_GluNa_2_ and 4, HTAB where a_1_, b_1_, c_1_, d_1_, a_2_, b_2_, c_2_, a_3_, b_3_, c_3_, a_4_, b_4_ and c_4_ represent the different phase transitions. Scan rate: 2°C min^-1^.

**Table S2.** Summarized results on the TGA of AAS, HTAB and their mixtures.

| **Surfactants** | **Temperature range/°C** | **%wt. remaining** |
| --- | --- | --- |
| C_12_MalNa_2_ | 80-105 | 89 |
|  | 105-186 | 79 |
|  | 247-310 | 68 |
|  | 351-412 | 25 |
| C_12_AspNa_2_ | 86-125 | 93 |
|  | 260-310 | 77 |
|  | 310-345 | 51 |
| C_12_GluNa_2_ | 73-123 | 96 |
|  | 330-406 | 82 |
|  | 422-508 | 55 |
| HTAB | 51-120 | 94 |
|  | 113-158 | 81 |
|  | 185-263 | 77 |
|  | 294-360 | 41 |
| C_12_MalNa_2_+HTAB* | 43-102 | 67 |
|  | 232-259 | 27 |
| C_12_AspNa_2_+HTAB* | 69-120 | 66 |
|  | 268-300 | 18 |
| C_12_GluNa_2_+HTAB* | 43-109 | 81 |
|  | 119-288 | 63 |

*AAS+HTAB=100 mM; AAS: HTAB= 40:60, M/M
